# Left ventricular hypertrophy and cardiac valve calcification and their impacts on survival in maintenance hemodialysis patients: A retrospective cohort study

**DOI:** 10.1097/MD.0000000000044181

**Published:** 2025-09-05

**Authors:** Xiaochun Wu, Zhiping Sun, Gang Yao, Fuhua Zhou

**Affiliations:** a Department of Critical Care Medicine, The Second Affiliated Hospital of Nanjing Medical University, Nanjing, Jiangsu Province, China; b Department of Nephrology, The Second Affiliated Hospital of Nanjing Medical University, Nanjing, Jiangsu Province, China.

**Keywords:** echocardiography, end-stage renal disease, hemodialysis, left ventricular hypertrophy, survival analysis, valve calcification

## Abstract

The purpose of this article was to study the distribution of left ventricular hypertrophy (LVH) and cardiac valve calcification (CVC), relevant factors, and the relationship of LVH and CVC with survival in maintenance hemodialysis (MHD) patients. A total of 281 MHD patients were included in this retrospective and follow-up study. Echocardiography measurements were performed to evaluate the left ventricular structure and cardiac valve. Left ventricular mass (LVM), LVM index, relative wall thickness, and prevalence of LVH and CVC were calculated. Factors related to LVH and CVC and patients’ death risk were analyzed. The primary outcome was death. The prevalence of LVH in this study was 50.53% (142 patients). Concentric hypertrophy, concentric remodeling, and eccentric hypertrophy were found in 39.86%, 25.98%, and 10.68% of patients, respectively. Factors associated with LVH included systolic pressure, CVC, hemoglobin, and calcium carbonate in a multivariate logistic regression model (all *P* <.01). The log-rank *χ*^2^, which were 10.957, 12.668, 10.181, and 8.474 at 6, 12, 18, and 24 months follow-up, respectively, in the Kaplan–Meier model demonstrated the lower survival rates in patients with LVH than in those without (all *P* <.01). The prevalence of CVC was 60.14% (169 patients). Aortic valve calcification, mitral valve calcification, or both were found in 54.09%, 33.10%, and 27.05% of patients, respectively. In a multivariate logistic regression model, the factors associated with CVC were coronary heart disease, LVH, calcium, age, dialysis age, and diastolic pressure (*P* <.05). In COX proportional hazard model, LVH resulted as an independent risk factor to all-cause death; the adjusted HR was 11.045, 4.382, 3.075, and 2.586 at 6, 12, 18, and 24 months follow-up, respectively (all *P* <.05). In MHD patients, LVH and CVC were highly prevalent, and LVH resulted as an independent risk factor for all-cause death.

## 1. Introduction

Left ventricular hypertrophy (LVH) and cardiac valve calcification (CVC) are 2 common cardiovascular comorbidities of end-stage renal disease (ESRD). According to the United States Renal Data Registry (USRDS) 2018, total cardiovascular disease prevalence of maintenance hemodialysis (MHD) and peritoneal dialysis reached 76.5% and 65.0%, respectively.^[1–3]^ China Dialysis Outcomes and Practice Pattern Study Phase 5 (DOPPS 5) showed that the total prevalence of cardiovascular disease in patients treated with dialysis exceeded 63%.^[4]^ The prevalence of LVH in MHD patients was reported to reach rates of 54.6% to 68.8%^[5,6]^ and even 68% to 75% in new dialysis patients.^[7,8]^ The prevalence of CVC in MHD patients was reported to be 58% to 76%^[9,10]^and even up to 65% in new dialysis patients.^[11]^

In MHD patients, LVH has been identified as an independent risk factor for death.^[12–14]^ Although a meta-analysis showed that CVC is a risk factor for cardiovascular and all-cause death in dialysis patients, few relevant studies exist.^[15]^ Meanwhile, nontraditional cardiovascular disease risk factors in the dialysis patients, such as uremia toxin, anemia, calcium and phosphorus metabolism disorder, and oxidative stress, increase the prevalence of cardiovascular disease. Also, the coexistence of these factors significantly increases adverse outcomes in hemodialysis patients.^[16–20]^ The relationship between LVH and CVC, i.e., their coexistence, has been detected in the early stage in dialysis patients.^[21]^ Then, the proportion of CVC in LVH patients increases significantly, especially in those with cardiac concentric hypertrophy.^[11]^ However, some studies also reported that eccentric cardiac hypertrophy in dialysis patients is correlated with valve calcification.^[22]^ Other studies suggested LVH was associated with aortic valve calcification (AVC).^[23]^

Transthoracic echocardiography is a simple and noninvasive examination used to assess cardiac structure and function. This study assessed ventricular thickness, cardiac chamber size, and valve calcification. In addition, the distribution of LVH and CVC and relevant factors in MHD patients were retrospectively analyzed in combination with their clinical data. We also examined the relationship between LVH and CVC, especially between LVH or CVC and all-cause death in Chinese patients.

## 2. Materials and methods

### 2.1. Study subjects

Patients receiving MHD treatment in the Blood Purification Center of the Second Affiliated Hospital of Nanjing Medical University between January 2010 and October 2013 were included in the study. They routinely received blood purification 3 times a week for 4 hours, including 2 times of dialysis and 1 time of diafiltration. Due to economic constraints, only dialysis treatment was given. All patients were given high flux dialyzer (ultrafiltration coefficient 44 to 49 mL/h/mm Hg), bicarbonate dialysis at dialysate flow of 500 ml/min and blood flow of 200 to 280 mL/min. The medical institution was a third-class A hospital, and the responsible medical staff had a blood purification qualification certificate. Inclusion criteria were the following: ESRD patients, age ≥18 years old, dialysis age >3 months, and patients who provided informed consent. Exclusion criteria were: underlying heart disease of hypertrophic or dilated cardiomyopathy, cardiac amyloidosis, complicated with a severe infection under investigation, and those who did not consent to blood tests or echocardiography. The ethics committee of the hospital approved the study.

### 2.2. Data collection and follow-up

Demographic data such as sex, age, weight, height, blood pressure, residual urine volume, daily activity and sun exposure, and smoking history were recorded. The eventual history of underlying kidney disease and dialysis, important comorbidities, and associated treatments, including renin-angiotensin system inhibitors, calcium carbonate, and active vitamin D preparations (calcitriol and alfacalcitol) were recorded referring to the hospital’s electronic medical records. Before dialysis, blood routine tests (automatic blood cell analyzer, Sysmex XS800), serum calcium and phosphorus, albumin (Hitachi 7170A automatic biochemical analyzer), and whole parathyroid hormone tests (DP 2000 automatic immunochemiluminescence analyzer in Nanjing Dian Medical Laboratory) were simultaneously performed in Clinical Laboratory. If dialysis was performed in the morning, the patient was asked to undergo a transthoracic echocardiogram in the afternoon, and if dialysis was performed in the afternoon or evening, the patient was asked to undergo a transthoracic echocardiogram the next morning. A 24-hour urine volume was divided into 3 grades: <100 mL, ≥100 mL and <400 mL, and ≥400 mL. Activity and light were divided into 2 grades, i.e., outdoor activity and sunlight exposure time of ≥2 hours and <2 hours per day.

As patients regularly came to the hospital for dialysis treatment, the changes in their condition were recorded each time in their medical records, combined with telephone follow-up. The primary endpoint event was death. All-cause death was defined as death from various causes, and the time and cause of death were recorded.

### 2.3. Echocardiographic evaluation

Color ultrasound (AGILENT SONOS 5500, HP) was equipped with a probe frequency of 2–4 MHz. Left ventricular mass (LVM) and LVM index (LVMI) were calculated according to the correction formula recommended by the American Society of Echocardiography.^[24,25]^ LVM (g) = 0.8 × 1.04 × (IVSTd [mm] + LVIDd [mm] + IVPWTd [mm])^[3]^-LVIDD [mm]^[3]^) +0.6, LVMI (g/m^2^) = LVM (g)/body surface area (BSA) (m^2^), DuBois formula^[26]^ was adopted for BSA (m^2^) = 0.20247 × height (m) ^0.725^ × weight (kg) ^0.425^. According to the recommendation of the American and European Society of Echocardiography, LVMI ≥ 125 g/m^2^ and ≥ 110 g/m^2^ are the diagnostic criteria for LVH for both men and women, respectively.^[24,25,27]^ According to the recommendation of the American Society of Echocardiography, relative wall thickness (RWT) of left ventricle ≥0.42 reflects the increase of myocardial thickness of left ventricle, RWT = (IVSTd + IVPWTd)/LVIDd.^[24,25]^ CVC was defined as bright echoes of >1 mm on one or more cusps of the aortic valve, mitral valve, or mitral annulus.^[25,28]^ The valve calcification was classified according to the site into 3 grades, i.e., double valve calcification, single valve calcification, and valve non-calcification.

### 2.4. Statistical analysis

SPSS18.0 software (IBM, New York) was used for statistical analysis. Measurement data were expressed as mean ± standard deviation (mean ± SD), and an independent sample *t*-test was used for between-group comparisons. Count data were expressed as rates (%), and the Chi-square test or corrected Chi-square test (χ2 test) was used for inter-group comparisons. Logistic regression was used for univariate and multivariate regression analysis and partial regression coefficient β test. Two-variable linear correlation analysis was used, with a correlation coefficient of Pearson r (r). Kaplan–Meier method was used for survival analysis (log-rank test). Cox proportional risk regression model was used for multivariate survival analysis. *P* <.05 represented statistical significance, with odds ratio (OR), hazard rate (HR), and confidence interval of 95% confidence interval.

## 3. Results

### 3.1. General conditions

A total of 281 MHD patients, including 169 males (60.14%) and 112 females (39.86%), with an average age of 60 ± 13 years (23–89 years), were enrolled. Underlying renal diseases included chronic glomerulonephritis (109 cases), hypertensive renal damage (67 cases), diabetic nephropathy (48 cases of type 2 diabetes, 2 cases of type 1 diabetes), polycystic kidney disease (12 cases), gout nephropathy (4 cases), chronic pyelonephritis (2 cases), obstructive nephropathy (2 cases), chronic interstitial nephritis (2 cases), and 33 cases of unknown etiology. A total of 268 patients were treated with blood purification 3 times per week, including 187 patients with bi-weekly hemodialysis and diafiltration, 81 patients with 3-weekly hemodialysis (without diafiltration), and 13 patients with bi-weekly hemodialysis. The mean dialysis age was 83 ± 65 months (6–342 months). Compared with females, male patients had a higher proportion of smoking, hypertension and gout, higher hemoglobin, and more dialysis dewatering volume, with no significant difference in other indicators (Table 1).

**Table 1:** Clinical data of 281 patients with maintenance hemodialysis.

Item	All patients	Male	Female	*t* or *χ*^2^	*P*
	(n = 281)	(n = 169)	(n = 112)		
Age (year)	60 ± 13	59 ± 14	60 ± 13	0.299	.765
BMI (kg/m^2^)	21.6 ± 3.1	21.8 ± 2.9	21.2 ± 3.4	1.450	.148
Smoking history, n (%)	76 (27.05%)	73 (43.20%)	3 (2.68%)	54.007	.000
Systolic pressure (mm Hg)	137 ± 18	138 ± 17	135 ± 19	0.962	.337
Diastolic pressure (mm Hg)	81 ± 9	81 ± 9	80 ± 9	0.726	.468
Mean arterial pressure (mm Hg)	99 ± 11	100 ± 10	99 ± 11	0.958	.339
Dialysis age (months)	83 ± 65	83 ± 65	81 ± 67	0.256	.798
Water removal per dialysis (kg)	2.5 ± 0.8	2.6 ± 0.8	2.3 ± 0.7	2.631	.009
Underlying disease of kidney disease					
Chronic glomerulonephritis, n (%)	109 (38.79%)	68 (40.24%)	41 (36.61%)	2.092	.719
Hypertensive kidney lesion, n (%)	67 (23.84%)	43 (25.44%)	24 (21.43%)		
Diabetic nephropathy, n (%)	50 (17.79%)	29 (17.16%)	21 (18.75%)		
Other, n (%)	22 (7.83%)	12 (7.10%)	10 (8.93%)		
Unknown, n (%)	33 (11.74%)	17 (10.06%)	16 (14.29%)		
Complication					
High blood pressure, n (%)	262 (93.24%)	162 (95.86%)	100 (89.29%)	4.615	.032
Diabetes, n (%)	65 (23.13%)	35 (20.71%)	30 (26.79%)	1.398	.237
Coronary heart disease, n (%)	34 (12.10%)	19 (11.24%)	15 (13.39%)	0.293	.588
Atrial fibrillation, n (%)	22 (7.83%)	12 (7.10%)	10 (8.93%)	0.312	.577
Gout, n (%)	30 (10.68%)	26 (15.38%)	4 (3.57%)	8.657	.003
Chronic obstructive pulmonary disease, n (%)	9 (3.20%)	7 (4.14%)	2 (1.79%)	0.566	.452
Cerebral infarction, n (%)	50 (17.79%)	30 (17.75%)	20 (17.85%)	0.001	.982
Drug use					
ACEI and ARB, n (%)	75 (26.69%)	48 (28.40%)	27 (24.11%)	0.635	.425
β receptor antagonist, n (%)	92 (29.37%)	58 (34.32%)	34 (30.36%)	0.480	.488
Calcium carbonate, n (%)	184 (32.74%)	114 (67.46%)	70 (62.50%)	0.732	.392
Vitamin D preparation, n (%)	163 (58.01%)	100 (59.17%)	63 (56.25%)	0.236	.627
Leukocyte (×10^9^/L)	5.95 ± 1.89	5.88 ± 1.89	6.04 ± 1.90	0.701	.484
Hemoglobin (g/L)	104 ± 17	106 ± 18	100 ± 15	2.783	.006
Serum albumin (g/L)	40.4 ± 5.0	40.3 ± 4.6	40.6 ± 5.4	0.554	.580
Calcium (mmol/L)	2.29 ± 0.29	2.28 ± 0.31	2.32 ± 0.27	1.139	.256
Corrected total calcium* (mmol/L)	2.28 ± 0.30	2.27 ± 0.30	2.30 ± 0.28	0.946	.345
Serum phosphorus (mmol/L)	1.64 ± 0.48	1.68 ± 0.48	1.57 ± 0.48	1.880	.061
Corrected calcium-phosphorus product (mmol2/L^2^)	3.74 ± 1.25	3.82 ± 1.21	3.64 ± 1.29	1.194	.233
Intact parathyroid hormone (pg/mL)	332 ± 561	370 ± 627	274 ± 438	1.414	.159

ACEI = angiotensin-converting enzyme inhibitor, ARB = angiotensin II receptor blocker.

* Corrected total calcium (mmol/L) = serum total calcium (mg/dl) + 0.08 × [40−albumin (g/L)] (calcium unit conversion 1mg/dL = 0.2495 mmol/L).

### 3.2. Distribution of LVM, LVMI and LVH

**Figure 1.**
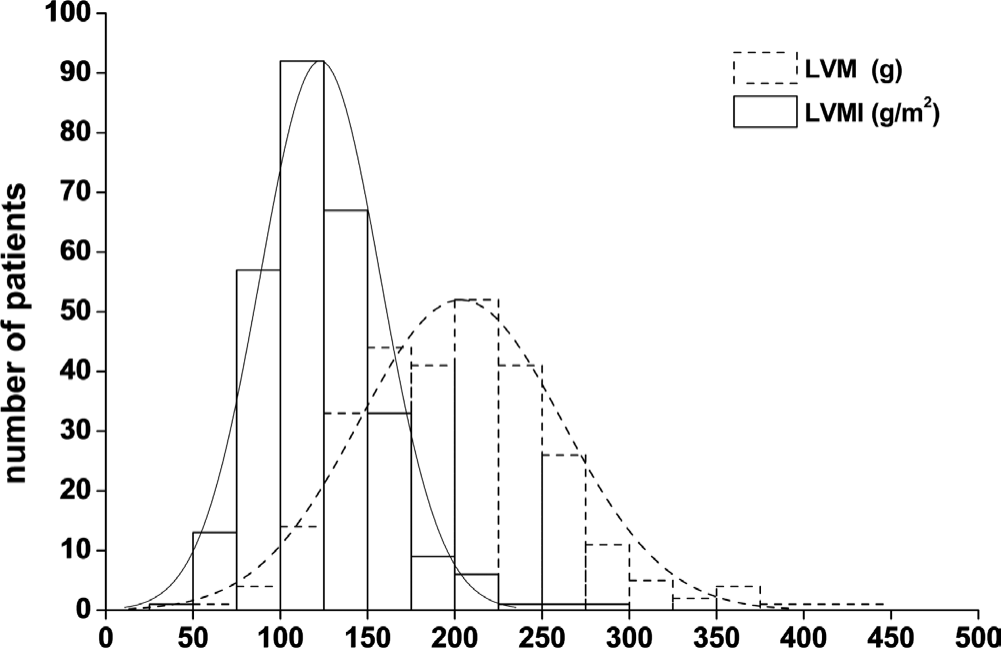
Left ventricular mass and left ventricular mass index in maintenance hemodialysis patients. LVM = left ventricular mass, LVMI = left ventricular mass index.

In this study, the mean LVM of 281 MHD patients was 203 ± 58 g (Fig. 1), 216 ± 56 g for men and 184 ± 58 g for women, t = 4.574, *P* = .000, with significant differences. The mean BSA was 1.73 ± 0.12 m^2^ for men and 1.54 ± 0.13 m^2^ for women. The mean LVMI was 123 ± 35 g/m^2^ (Fig. 1), 125 ± 32 g/m^2^ for men, and 120 ± 39 g/m^2^ for women (T = 1.027, *P* = .305), without significant difference. A total of 142 patients (50.53%) were diagnosed with LVH, including 77 (45.56%) out of 169 male patients and 65 (58.04%) out of 112 female patients. Chi-square test χ2 = 4.193, *P* = .041 showed a significant difference in the prevalence of LVH between men and women.

### 3.3. Analysis of factors correlated with LVH

**Figure 2.**
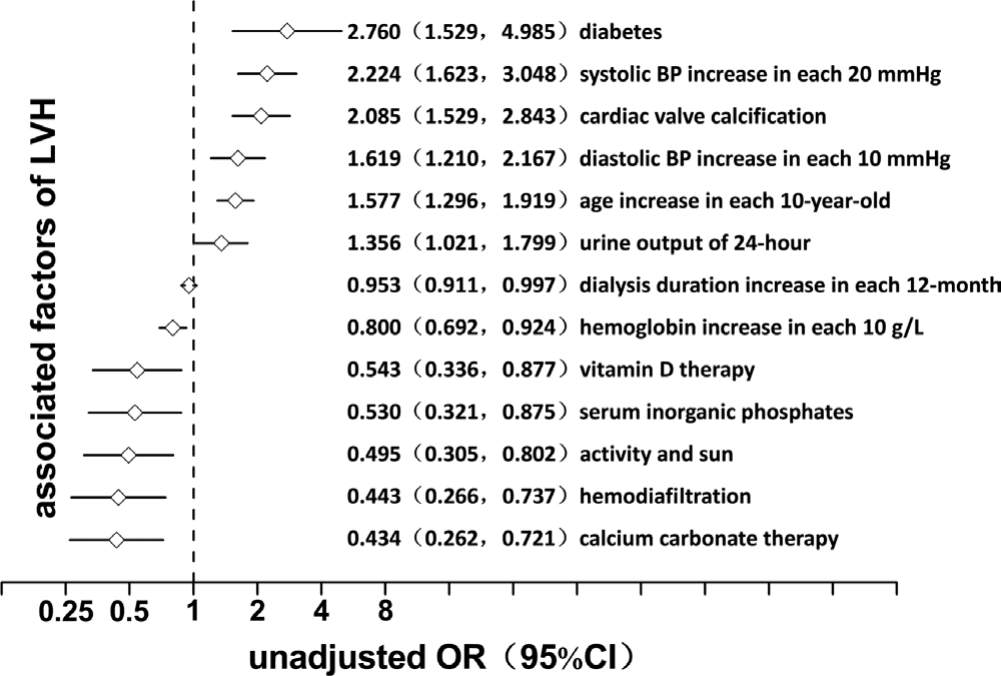
Univariate logistic regression analysis for left ventricular hypertrophy in maintenance hemodialysis patients. CI = confidence interval, LVH = left ventricular hypertrophy, OR = odds ratio.

**Figure 3.**
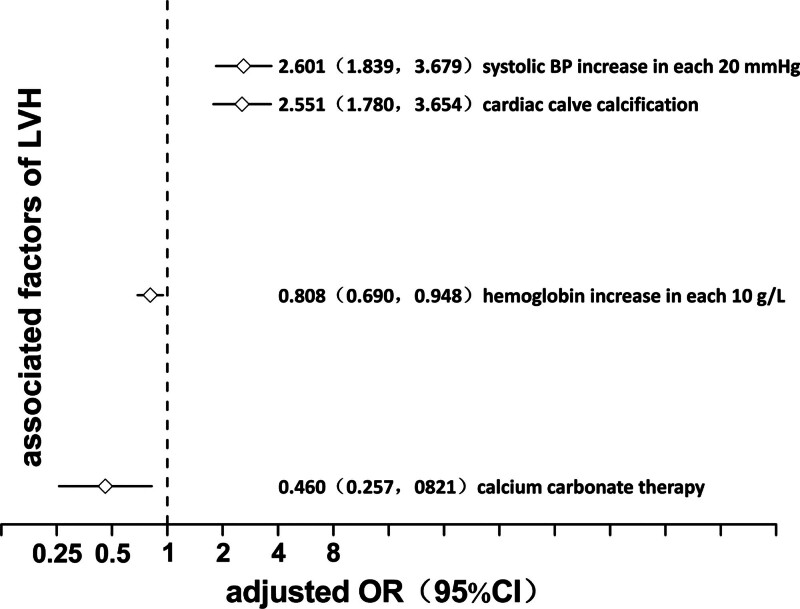
Multivariate logistic regression analysis for left ventricular hypertrophy in maintenance hemodialysis patients. CI = confidence interval, LVH = left ventricular hypertrophy, OR = odds ratio.

A logistic regression model was used to analyze the factors correlated with LVH. In the univariate model, in descending order of OR value, the factors associated with LVH included diabetes mellitus, systolic blood pressure, CVC, diastolic blood pressure, age, 24-hour urine volume (residual urine volume), dialysis age, hemoglobin, vitamin D, inorganic phosphorus, activity and light, hemodiafiltration, and calcium carbonate (*P* = .001, 0.000, 0.000, 0.001, 0.000, 0.035, 0.036, 0.002, 0.013, 0.013, 0.004, 0.002, and 0.001, respectively). ORs of the first 6 factors were >1, suggesting that the advantage of LVH increased with the increase in value. Conversely, OR of the last 7 factors was <1, suggesting that the advantage of LVH decreased with the value increase (Fig. 2). In the multivariate model, the factors of LVH were systolic blood pressure, CVC, hemoglobin, and calcium carbonate in descending order of OR value (*P* values = .000, .000, .009, and .009, respectively). ORs of the first 2 factors were >1, suggesting that LVH advantage increased with the value increase. Conversely, ORs of the last 2 factors were <1, suggesting that the advantage of LVH decreased with the value increase (Fig. 3).

### 3.4. Left ventricular geometry analyzed by LVMI and RWT

**Figure 4.**
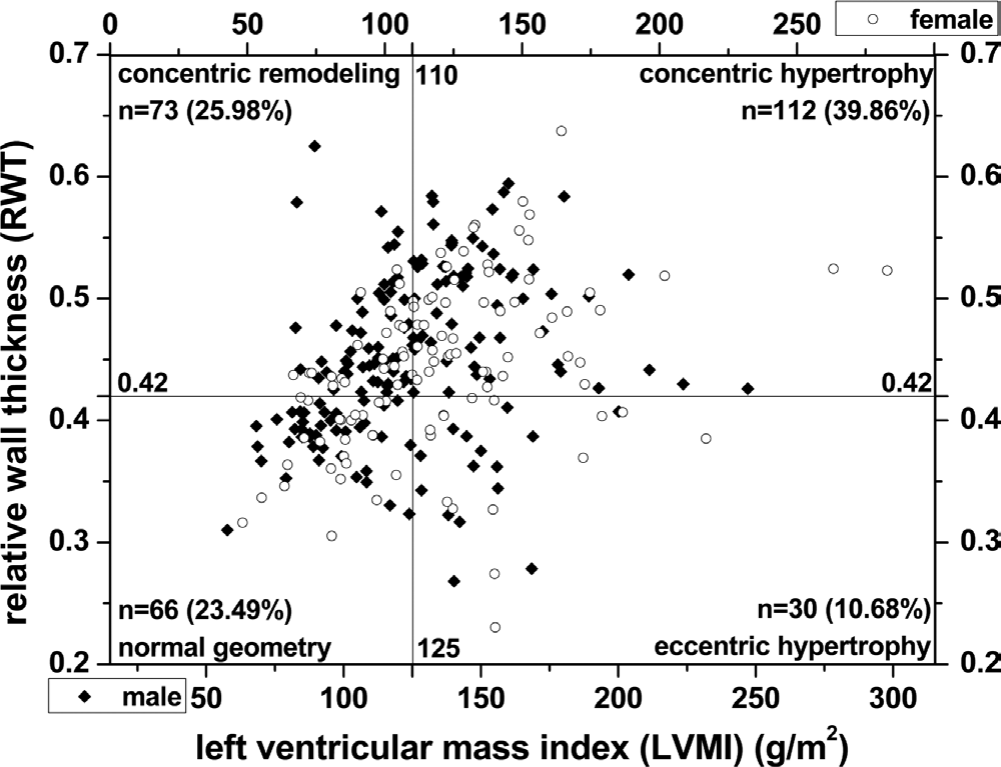
Relationship between the left ventricular mass index and relative wall thickness in maintenance hemodialysis patients.

In this study, 185 of 281 MHD patients had increased RWT (≥ 0.42), accounting for 65.84%. Two-variable linear correlation analysis suggested *R* = 0.324, *P* = .000 between LVMI and RWT. After a comprehensive analysis of LVMI and RWT, 39.86% of the patients showed concentric hypertrophy (112 cases), defined as increased LVMI and RWT. This LVH was mainly caused by left ventricular cardiac hypertrophy, while 10.68% of the patients had eccentric hypertrophy (30 cases), which indicated that LVMI increased, but RWT remained normal. An enlarged left ventricular cardiac chamber mainly caused this LVH. There were 25.98% of patients with concentric remodeling (73 cases), i.e., LVMI was normal, but RWT increased. These patients did not meet the diagnostic criteria for LVH, but their left ventricular myocardial thickness increased, and only 23.49% had normal left ventricular geometry (66 cases) (Fig. 4).

### 3.5. Distribution of CVC

**Figure 5.**
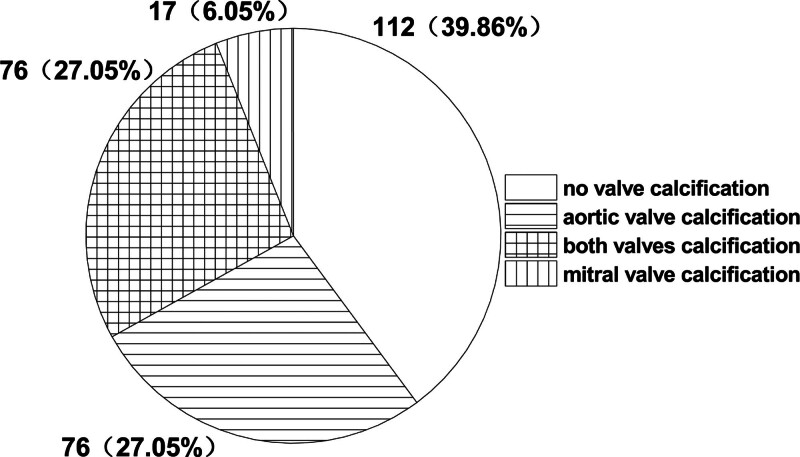
Cardiac valve calcification in maintenance hemodialysis patients.

In this study, 60.14% of patients (169 cases) diagnosed by chest wall echocardiography had CVC, 54.09% of patients (152 cases) had AVC, 33.10% of patients (93 cases) had mitral valve calcification (MVC), including 76 patients (27.05%) with simultaneous calcification of both aortic and mitral valve (bivalve calcification), and 39.86% patients (112 cases) without valve calcification (Fig. 5).

### 3.6. Analysis of CVC correlation factor

**Figure 6.**
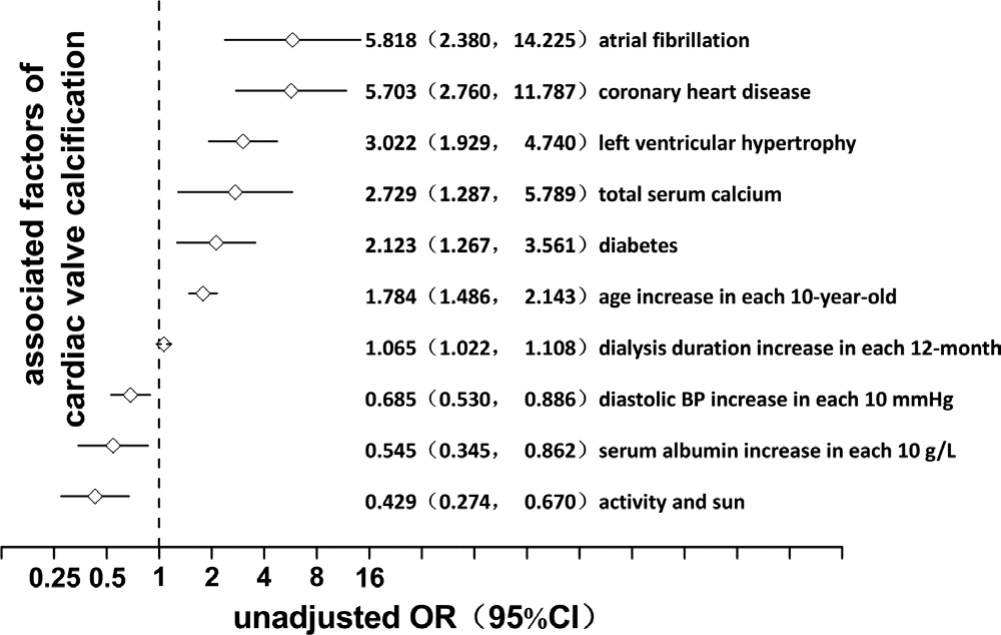
Univariate Logistic regression analysis for cardiac valve calcification in maintenance hemodialysis patients. CI = confidence interval, CVC = cardiac valve calcification. OR = odds ratio.

**Figure 7.**
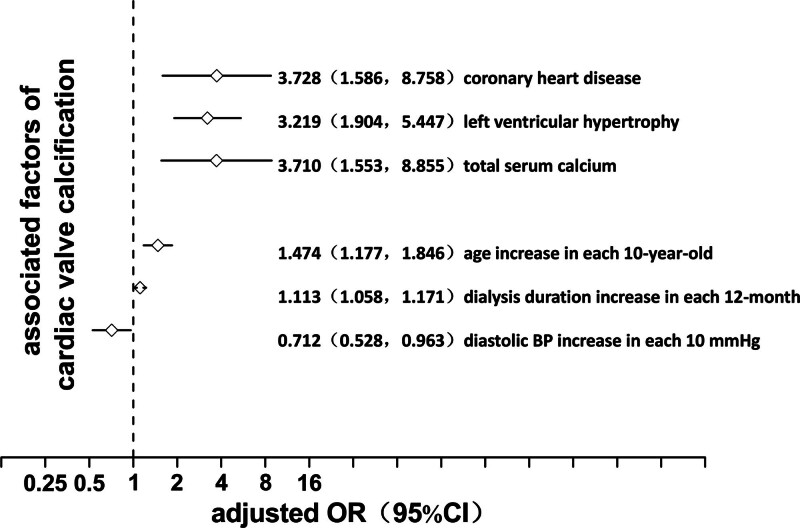
Multivariate logistic regression analysis for cardiac valve calcification in maintenance hemodialysis patients. CI = confidence interval, CVC = cardiac valve calcification, OR = odds ratio.

A logistic regression model was used to analyze the correlation factors of CVC for univariate and multivariate analysis. According to the number of valve calcifications, 39.86% of patients (112 cases) had no valve calcification, 33.10% of patients (93 cases) had single valve calcification, and 27.05% of patients (76 cases) had double valve calcification. In the univariate regression model, the factors of CVC included atrial fibrillation, coronary heart disease (CHD), LVH, blood calcium, diabetes, age, dialysis age, diastolic blood pressure, albumin, activity, and light exposure (*P* values = .000, .000, .000, .009, .004, .000, .003, .004, .009, and .000, respectively). The factors with regression coefficient (β) >1, i.e., the higher level, indicated a greater susceptibility to heart valve calcification. In descending order of OR, under a history of atrial fibrillation, the OR of double valve calcification versus single valve calcification and no valve calcification OR heart valve calcification versus no valve calcification was 5.818. Under a history of CHD, the OR was 5.703. Under LVH, the OR was 3.022; OR was 2.729 for each increase of 1 mmol/L blood calcium. The OR was 2.729 for each increase of 1 mmol/L of blood calcium, 2.123 for diabetes history, 1.784 for each 10 years increase in age, and 1.065 for each 12 monthly increase in dialysis time. Factors with regression coefficient (β) <1 included diastolic blood pressure, albumin, activity, and light, and the OR of valve calcification was 0.685 for every 10 mm Hg increase in diastolic blood pressure, i.e., 1/0.685 = 1.460 for every 10 mm Hg decrease in diastolic blood pressure and 0.545 for every 10 g/L increase in albumin. Otherwise, it was 1/0.545 = 1.835. OR of activity and light was 0.429; otherwise, it was 1/0.429 = 2.331 (Fig. 6). In the multivariate regression model, under a history of CHD, OR was 3.728 (*P* = .003) for valve calcification, 3.219 (*P* = .000) for LVH, 3.710 (*P* = .003) for every 1mmol/L increase in blood calcium, 1.474 (*P* = .001) for every 10 years increase in age, 1.113 (*P* = .000) for each additional 12 months of dialysis age, 0.712 (*P* = .027) for each additional 10 mm Hg of diastolic blood pressure, 1/0.712 = 1.404 for each additional 10 mm Hg of diastolic blood pressure, respectively (Fig. 7).

### 3.7. Kaplan–Meier survival analysis of patients with all-cause death

The average follow-up time of 215 patients was 16 ± 6 months (2–24 months), and 29 patients died during the follow-up. The direct cause of death for 15 patients was cardiovascular disease, accounting for 51.72% of the total number of deaths, including 6 cases of cerebral hemorrhage, 5 cases of sudden cardiac death, 2 cases of pulmonary embolism, 1 case of heart failure, and 1 case of cerebral infarction. In addition, 14 patients died due to severe infections, severe trauma, unknown reasons, etc.

**Figure 8.**
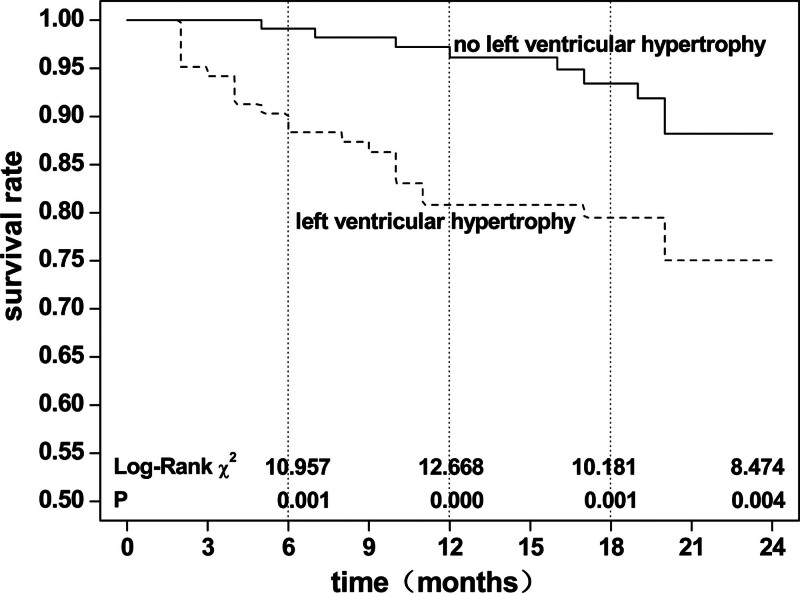
Kaplan–Meier survival analysis for all-cause death in maintenance hemodialysis patients according to left ventricular hypertrophy.

**Figure 9.**
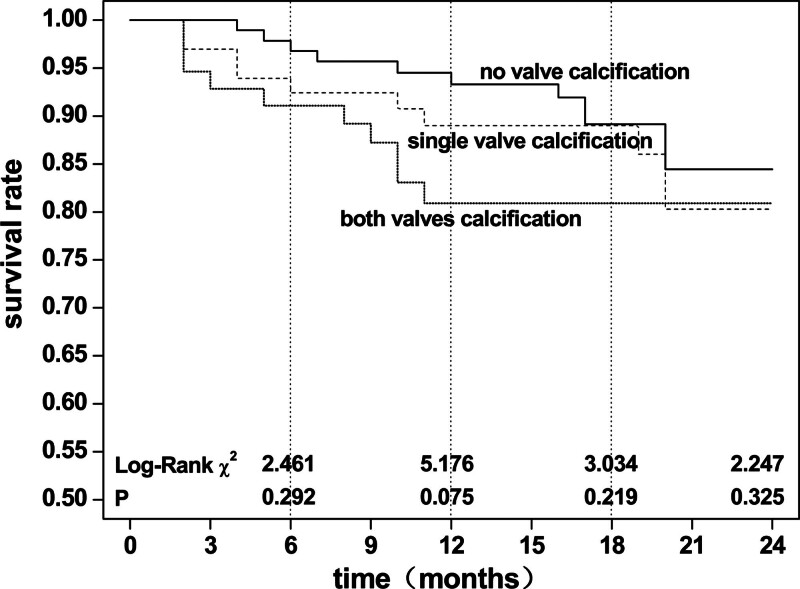
Kaplan–Meier survival analysis for all-cause death in maintenance hemodialysis patients according to cardiac valve calcification.

There were 21 patients with LVH (14.79%) and 8 without LVH (5.76%). The chi-square test was χ2 = 6.193, *P* = .013, indicating a significant difference in mortality between the 2 groups. There were 10 patients with double valve calcification (13.16%), 9 patients with single valve calcification (9.68%), and 10 patients with no valve calcification (8.93%). The chi-square test was χ2 = 0.937, *P* = .626, revealing no significant difference in mortality between the 3 groups. Kaplan–Meier survival analysis (Log-Rank test) showed that non-LVH patients had higher survival rates than LVH patients (*P* <.05), At 6 months, χ2 = 10.957, *P* = .001, 12 months, χ2 = 12.668, *P* = .000, 18 months, χ2 = 10.181, *P* = .001, and 24 months follow-ups, χ2 = 8.474, *P* = .004 (Fig. 8). There was no significant difference in survival rate between patients with different grades of valve calcification (*P*> .05); however, the survival curve showed a lower survival rate in patients with valve calcification than in those without (Fig. 9).

### 3.8. Cox proportional risk regression analysis of all-cause death

**Figure 10.**
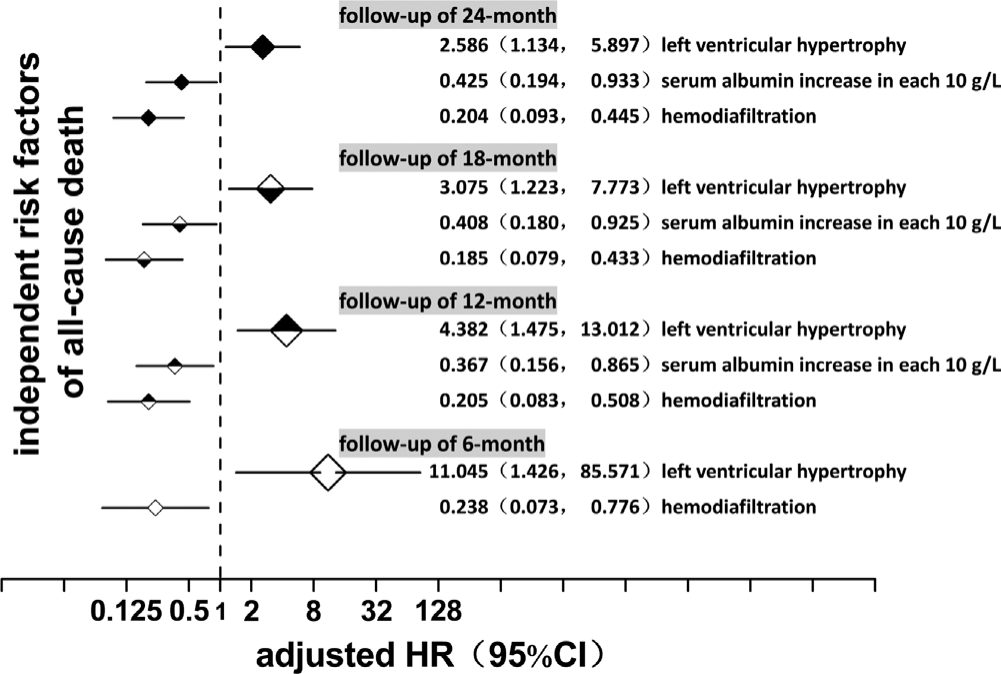
Cox proportional hazards regression analysis for all-cause death in maintenance hemodialysis patients. CI = confidence interval, HR = hazard rate.

Cox proportional hazards regression model was used to analyze 215 patients included in the follow-up multivariate survival analysis. The variables included LVH, albumin, hemodiafiltration, age, dialysis age, activity and sun exposure, cerebral infarction, diabetes, CHD, hemoglobin, blood phosphorus, ejection fraction, vitamin D, calcium carbonate, and heart valve calcification. Our results showed that LVH was an independent risk factor for all-cause death at multiple time points during the study follow-up period (adjusted HR >1, *P* <.05). Adjusted HRs were 11.045 (*P* = .021) (6 months), 4.382 (*P* = .008) (12 months), 3.075 (*P* = .017) (18 months), and 2.586 (*P* = .024) (24 months), respectively. The protective factors of all-cause death were increased serum albumin and regular hemodialysis and filtration treatment (adjusted HR <1, *P* <.05). CVC was not an independent risk factor of all-cause death (*P* >.05) (Fig. 10).

## 4. Discussion

MHD is regarded as one of the main life-sustaining treatments for ESRD patients. Thanks to the development of medical technology, life quality has been continuously improving; however, cardiovascular disease is still the first cause affecting the prognosis of dialysis patients.^[12–20]^ The early cardiovascular clinical practice guidelines for kidney disease outcomes quality initiative have pointed out that LVH and CVC, 2 cardiovascular comorbidities, are highly prevalent and are associated with adverse outcomes, thus gaining increasing interest among researchers.^[29]^ The present study observed the distribution and associated factors of LVH and CVC in MHD patients, studied the correlation between LVH and CVC, further analyzed the characteristics of left ventricular geometry in dialysis patients, and conducted an all-cause death survival analysis of such patients.

In the present study, the prevalence of LVH reached 50.53%, and the prevalence of female patients was slightly higher than that of male patients, without significant differences. The difference in LVM was statistically significant, while that in LVMI was not because of the large difference in weight and height between genders. The high prevalence of LVH is similar to the results reported in many publications, suggesting that LVH is common in dialysis patients, accounting for 54.6% to 68.8%.^[5,6,14]^ In this study, LVMI and RWT were combined to analyze the left ventricular geometry and cardiac structure was classified into concentric hypertrophy (39.86%), eccentric hypertrophy (10.68%), concentric remodeling (25.98%) and normal geometry (23.49%). Distinguishing between 2 types of LVH (concentric LVH and eccentric LVH), which have been reported to manifest at different stages of disease progression, has an important clinical value. When cardiac pressure and volume load increase, left ventricular concentric hypertrophy first occurs to enhance myocardial contractility, subsequently impairing diastolic function and eventually leading to left ventricular concentric hypertrophy, enlargement of the heart cavity and total heart failure.^[30,31]^ While there are few studies on dialysis patients, relevant reports on non-dialysis patients can be found. In 2009, a European study of patients with stage 3 to 4 chronic kidney disease showed that LVH accounted for 47.01% of patients, including 18.85% of patients with concentric hypertrophy, 28.16% of patients with eccentric hypertrophy, and 15.30% of patients with concentric remodeling.^[32]^ Another European and American study, which was conducted in 2016 and included patients with stage 2 to 5 chronic kidney disease, showed that LVH accounted for 55.95%, including 28.09% of concentric hypertrophy, 27.87% of eccentric hypertrophy, and 15.96% of concentric remodeling. With the progress of renal function, the prevalence of concentric and eccentric hypertrophy has been increasing.^[33]^

In this study, the prevalence of CVC was 60.14%, among which the prevalence of AVC was 54.09%, MVC was 33.10%, and double valve calcification accounted for 27.05%. Relevant literature also showed that the prevalence of CVC was high in dialysis patients.^[9,10]^ As for the calcification of different valves, there are similar reports. In 2011, a study in the United States detected AVC and MVC in 44.4% and 38.2% of dialysis patients.^[34]^ In 2012, a study from Japan showed that AVC and MVC were found in 92.68% and 100% of patients with >10 years of dialysis,^[35]^ indicating that valve calcification was related to dialysis age. Different degrees of CVC were also found in peritoneal dialysis patients; however, the prevalence was lower than in hemodialysis patients. E.g., a study from Hong Kong in 2003 showed that CVC was found in 32.29% of peritoneal dialysis patients and AVC and MVC in 17.18% and 22.40% of peritoneal dialysis patients, respectively,.^[36]^ A study conducted in China Macao in 2009 reported that CVC in 24.78% of peritoneal dialysis patients, while AVC and MVC accounted for 13.04% and 16.52%, respectively.^[37]^ Therefore, whether different dialysis methods affect the rate of valve calcification and underlying causes should be further discussed.

During the follow-up, it was found that 51.72% (15 cases) of all 29 death patients died of cardiovascular disease, indicating that the disease is still an important cause of death in dialysis patients. Kaplan–Meier survival analysis revealed that compared with other patients, LVH and CVC patients had lower survival probability (the former was statistically significant, while the latter was not). Cox proportional risk regression analysis found that LVH was an independent risk factor for all-cause death after adjustment for multiple factors, and LVH was the primary factor. Analyses at several follow-up time points showed the same pattern. In 2017, a multi-center follow-up cohort study of patients with ESRD in Italy and France showed that LVMI was an independent risk factor for all-cause and cardiovascular deaths.^[14]^ However, different opinions were also proposed. A systematic review of the American Journal of Kidney Nephrology (AJKD) in 2016 showed that the changes in all-cause and cardiovascular deaths were not completely consistent after the LVH improvement caused by the intervention.^[38]^ Therefore, further stratified follow-up observation is recommended. The relationship between CVC and the prognosis of patients has also been reported. A 2011 American follow-up study with an average of 5 years identified MVC as an independent risk factor for all-cause mortality in patients.^[34]^ A follow-up study from Japan conducted in 2009 revealed that AVC was a predictor of in-stent restenosis in dialysis patients and was associated with cardiovascular death.^[39]^ Valve calcification is a type of cardiovascular calcification often found in patients with CHD and carotid atherosclerosis. The relationship between several diseases and death needs to be more objectively distinguished in studies.^[40]^ In the present study, we also found that the survival probability of patients with valve calcification was low. However, the difference did not reach statistical significance after hypothesis testing, which may be related to the sample size of patients, selection bias, and other factors.

LVH and CVC in dialysis patients were found to be mutually related factors. Although the causal relationship could not be established, our results still suggest that positive control of 1 disease might benefit the control of the other. However, there are few reports on the relationship between these conditions. In a study of peritoneal dialysis patients conducted in Turkey in 2012, LVH in CVC patients accounted for 62% (36% in non-CVC patients), and CVC in LVH patients accounted for 42% (20% in non-LVH patients).^[41]^ A study conducted in Japan in 2017 showed that among hemodialysis patients with CVC and coronary artery calcification, the proportion of LVH and CVC was 96.4% and 77.5%, respectively, among which the proportion of CVC was higher in patients with concentric hypertrophy.^[11]^ Therefore, further stratification studies are needed to clarify the relationship and co-pathogenic factors.

Yet this study has a few limitations. This descriptive retrospective study aimed to analyze and explore related diseases’ risk factors and etiology. No strict control and intervention design may lead to bias. For example, the analysis of disease-related factors showed a variety of related factors, and the analysis was complicated. Future prospective control and intervention studies with bigger sample sizes and multi-center cooperation are needed.

In conclusion, we found that the prevalence of LVH and CVC in MHD patients was high besides being correlated. The survival probability of LVH patients was lower than that of non-LVH patients, and LVH was an independent risk factor for all-cause death in these patients. Therefore, making integrated diagnosis and treatment for dialysis patients is very important. Besides dialysis methods, drug therapy, underlying disease control, and avoidance of comorbidity, lifestyle, exercise, diet, psychology, and other factors should be given adequate attention. Also, primary and secondary prevention should be highly emphasized to effectively improve dialysis patients’ quality of life and prognosis.^[42]^

## Author contributions

**Conceptualization:** Xiaochun Wu, Zhiping Sun, Gang Yao, Fuhua Zhou.

**Data curation:** Xiaochun Wu, Zhiping Sun, Gang Yao, Fuhua Zhou.

**Formal analysis:** Xiaochun Wu, Zhiping Sun, Gang Yao, Fuhua Zhou.

**Investigation:** Xiaochun Wu, Zhiping Sun, Gang Yao, Fuhua Zhou.

**Methodology:** Xiaochun Wu, Zhiping Sun, Fuhua Zhou.

**Project administration:** Fuhua Zhou.

**Supervision:** Fuhua Zhou.

**Writing – original draft:** Xiaochun Wu, Zhiping Sun, Gang Yao, Fuhua Zhou.

**Writing – review & editing:** Xiaochun Wu, Zhiping Sun, Gang Yao, Fuhua Zhou.
